# The Rheumatologist’s Role in the Battle Against COVID-19: Insights from the Front Line and Challenges for the Future

**DOI:** 10.31138/mjr.31.3.247

**Published:** 2020-09-21

**Authors:** Christos Koutsianas, Katerina Chatzidionysiou

**Affiliations:** 1 Department of Rheumatology, The Dudley Group NHS Foundation Trust, Dudley, West Midlands, United Kingdom; 2 Rheumatology Unit, Department of Medicine, Karolinska University Hospital, Solna, Karolinska Institute, Stockholm, Sweden

**Keywords:** Rheumatic diseases, rheumatology, COVID-19, SARS-CoV-2, telemedicine, hyper-inflammation

## Abstract

As the worldwide burden of COVID-19 increases exponentially, healthcare systems are plagued by unprecedented pressure. In this setting, many rheumatologists across the globe have been recruited to support the front line, facing several unexpected challenges, but also providing valuable skills in combating COVID-19. At the same time, the rheumatic disease patient population may be especially vulnerable to such a rapidly contagious infectious disease and thus needs care and support that has to be provided quickly and efficiently. Clear advice on viral spread mitigation, precise guidelines on immunosuppressive treatment use and alternative methods of providing care, such as telemedicine, are a few of the rheumatologists’ new challenges in caring for their patients in the COVID-19 era. Finally, among other specialties, rheumatologists hold a unique place in the fight against the hyper-inflammatory state caused by severe SARS-CoV-2 infection, leading to increased morbidity and mortality. Given their vast experience in the use of biologic and targeted therapies, rheumatologists should lead the way in developing reliable scientific evidence for the optimal treatment of severe COVID-19.

## INTRODUCTION

At the end of 2019, the rheumatology community was looking into the future very optimistically, standing at the dawn of a new decade full of promises of new potent medication, precision medicine and deeper understanding of the pathophysiological mechanisms causing rheumatic disease. As the initial reports of a cluster of cases of fatal bilateral pneumonia of unknown aetiology came from the Wuhan province of China,^1^ little did we suspect that severe acute respiratory syndrome coronavirus 2 (SARSCoV-2) was going to drastically change our lives and the world as we knew it. The Coronavirus disease 2019 (COVID-19) pandemic has since acquired unexpected dimensions, posing the most challenging health crisis for a generation with almost 3 million confirmed cases and more than 200,000 deaths worldwide at the end of April 2020^2^. It has affected without discrimination the personal and professional lives of people across the globe with several physical, psychological, socioeconomic and political complications. The rapid spread of severe COVID-19 cases puts extraordinary pressure in national healthcare systems and has been testing their capacity limits. The shortage of ventilators and adequate intensive care unit (ICU) beds has been a cause for anxiety, but perhaps the biggest challenge is the presence of a healthy workforce able to deal with the management of the pandemic, especially since the demand caused by critically ill patient surges may last for several months and the levels of medical and allied health professional (AHP) staff absence are expected to be very high.

In this setting, rheumatologists are specialists who can offer valuable skills in combating COVID-19. Up until now, they have done so willingly and without hesitation. An integrated preparation and action plan for the rheumatologist should focus in three main key areas: ensuring the sustainability of the healthcare setting by supporting the front line, maintaining care of high quality for the vulnerable rheumatic patient population and offering valuable expertise in the molecular mechanisms and use of anti-rheumatic medication in the treatment of SARSCoV-2 infection.

## PERSPECTIVES FROM THE FRONT LINE

As the tragic news of this pandemic’s impact came from countries like China, Italy and Spain, it became clear that forward emergency planning to expand treatment capacity was of the utmost importance in all other areas. Anticipating the impact on the hospitals’ capacity and resources, as well as the dwindling numbers of capable front-line healthcare personnel due to illness, reallocation of staff and repurposing of wards was warranted. Both in Sweden and the UK, redeployment of secondary care specialist staff was one of the earliest healthcare systems preparatory actions. In this context and in the midst of the anxiety of anticipation, rheumatologists were very quickly reallocated in emergency rooms, general internal medicine and respiratory wards and step-down settings, providing care for COVID-19 positive patients.

Being on the front-line during these unprecedented times causes a flurry of contrasting thoughts and emotions. The need for stepping up in such a health crisis reaches back to the ancient ‘Hippocratic oath’ and the calling of medics to provide help to people. It fills you with a sense of duty, passion for providing exemplary patient care when it is most needed and enthusiasm to support your hospital team. On the other hand, there are several opposing forces stemming from the anxiety for the unknown, the intimidation of working in unfamiliar territory and out of one’s comfort zone and the fear for personal health, but also for transmitting the virus to family and loved ones. At the same time, fighting against an up until recently unencountered enemy can be extremely challenging. Healthcare workers face excessive workloads and experience uncertainty in their decision making, which has to take place amidst a huge amount of clinical information and a ‘boom’ in the relevant medical literature, which constantly and rapidly updates and is frequently of doubtful credibility and quality.^3,4^ Also, rheumatologists, much like other secondary care specialists chiefly dealing with outpatients, are not accustomed to making end-of-life decisions in their routine work life and can thus find themselves in front of difficult moral dilemmas, causing further anxiety in their practice.

All these sources of anxiety need to be identified and addressed early on and rheumatologists will need to be supported in these difficult times. Early deployment with opportunities for ‘shadowing’ more experienced staff is paramount in order to achieve the right induction, training and familiarity with their new role. The hospital leadership should be able to provide adequate personal protective equipment and rapid access to testing for their staff, maintain channels of input and feedback, support the physical, psychological and emotional needs and ensure proper indemnity coverage.^5^ In these settings, building competence and confidence is an important team-based, rather than individual, capability. In the experience of the authors, collaboration and team working with other staff groups and allied health professionals can lead to an unmatched sensation of comradery and the best way to manage one’s own fears and anxiety. Regular communication, altruism and solidarity against the common enemy can turn the tide and aid in the provision of appropriate clinical care. Finally, rheumatologists should keep in mind that as a specialty we are familiar with a thorough, systematic and holistic review of our patients, thus being able to provide precious general medicine skills.

## CARING FOR THE RHEUMATIC DISEASE POPULATION

This particularly challenging, and at times, war-like situation, completely changes the routine practice of rheumatology. As principal as it is to be supportive of the front-line, it is equally important to care for our rheumatic disease patient population, which in the wake of a rapidly contagious infectious disease may be especially vulnerable, given our patients’ age, comorbidities, underlying conditions and type of treatments used.

As the relationship between inflammation and infection is very complex, the interaction of SARS-CoV-2 and rheumatic disease can also be multifaceted.^6^ First of all, it is well recognised that infections can contribute to flares of pre-existing rheumatic disease. Also, as is the case with other viruses (eg, chikungunya, parvovirus, etc.), coronaviruses have been linked to triggering not only polyarthritis-like syndromes, but also increased incidence of rheumatoid arthritis.^7^ Furthermore, patients with rheumatic disease appear to be in a higher risk for developing infection compared to the general population. This risk may be dictated by a sensitive balance between the levels of disease activity (which, if high, may be blunting the immune system’s response) and the potency of the iatrogenic immunosuppression used. All of the above need to be taken into consideration when assessing the risk for severe SARS-CoV-2 infection in patients with rheumatic diseases. Up until now, rheumatic disease has not been identified as one of the comorbidities linked to higher COVID-19 related mortality. The relevantly low incidence of rheumatic patients with severe COVID-19 has been noted in the medical literature,^8,9^ and this is corroborated by the authors’ experience. Whether this should be attributed to an effect of the use of disease modifying anti-rheumatic drugs (DMARDs) in preventing cytokine release syndrome (CRS) that has been identified as the major contributor to acute respiratory distress syndrome (ARDS) or it is a result of our patients’ awareness and prompt adoption of social distancing and shielding behaviour remains to be elucidated.

In any case, it is the duty of the rheumatologists to care for, advice and support their patients to the best of their knowledge in these challenging times. An effort to create a global registry for rheumatic patients afflicted by COVID-19^10^ was fruitful within mere days and is expected to provide significant help in addressing the information deficit using both physician and patient derived data. At amazing speed, national and international societies published guidelines for the management of patients with rheumatic disease^11^ in the face of the COVID-19 pandemic. These stress the importance of social distancing and other viral spread mitigation recommendations, but also urge patients not to discontinue their DMARD treatment without such counselling by their physicians. The latter is especially true for corticosteroid therapy, where abrupt cessation can cause more harm through adrenal insufficiency.

As expected, patient anxiety caused by the virus outbreak posed an enormous workload at the initial point of contact in our rheumatology departments. We used several strategies to manage this increased demand, including pre-emptively identifying patients deemed of being at higher risk and directly contacting them for instructions, signposting to departmental and government advice on our rheumatology helplines and websites and regularly updating the relevant recommendations. Nevertheless, perhaps the most important aid for our patients is that of an empathetic listening ear to receive and entertain their concerns during these stressful and uncertain times; physicians, clinical nurse specialists and secretaries made a point of being available to achieve this end. Finally, one of the recommendations to help in ascertaining patient safety in this setting is the avoidance of routine face-to-face consultations. To this end, it is necessary to have the ability to provide remote consultations. The use of telemedicine in rheumatology has been contemplated in recent years and, even though it was met with some hesitation due to the lack of physical examination, there is data that it can be an acceptable alternative for the follow up of patients with established rheumatic disease.^12^ Furthermore, EULAR has recently advocated the use of mobile health applications in order to aid self-management of musculoskeletal disease.^13^ In the effort to implement strict social distancing and isolation of vulnerable individuals, we have swiftly adopted remote (telephone or video) consultations for our outpatient follow up visits. It quickly became clear that a lot of patients could be managed effectively from a distance. Telemedicine offered us a valuable service to enable the triage of new non-urgent referrals, to be able to evaluate known patients and provide meaningful advice. The necessary infrastructure was very quickly in place and there was no significant resistance in its adoption by patients and care providers alike, especially after a short period of orientation. The authors are certain that telemedicine will prove extremely valuable in the long term, even when the current pandemic subsides. The current positive experience will hopefully serve as a guide for further implementation.

## THE RHEUMATOLOGIST’S ROLE IN COMBATING COVID-19: BLOCKING THE CYTOKINE STORM

After an initial symptomatology of a viral-like illness with fever, respiratory symptoms and malaise, a subset of COVID-19 infected patients will develop a severe acute respiratory distress syndrome, considered to be driven by the host’s immune system and characterized by hyperinflammation. As such, the rheumatologist, being perhaps no expert on viruses, but holding a deep knowledge and expertise on the immune system, may have a pivotal role in managing COVID-19 not only in patients with rheumatic disease but also in the general population.^14,15^

Similar hyperinflammatory conditions, such as macrophage activation syndrome, are seen in the context of some rheumatological disorders (eg, adult-onset Still disease, systemic juvenile idiopathic arthritis, systemic lupus erythematosus). Both adult and paediatric rheumatologists are the specialists with the largest experience in this field. Early after the outbreak of COVID-19, the potential role of immunomodulatory treatment in severe ARDS became the focus of ongoing research in parallel to the that of anti-viral treatment. Many cytokines, such as interleukin (IL)-1, IL-6, IL-18, tumour necrosis factor (TNF) and interferon (IFN)-γ, have a pivotal role in the cytokine storm seen in severe COVID-19. From an immunological perspective, the severity of COVID-19 seems to correlate with increased levels of several cytokines. Potent drugs that block some of the above cytokines are routinely used for the treatment of diverse rheumatological conditions. Thus, rheumatologists have acquired significant experience in their administration and management.

Tocilizumab, an anti-IL6 receptor monoclonal antibody approved for the treatment of rheumatoid arthritis and giant cell arteritis, quickly improved some of the clinical manifestations of COVID-19, such as fever and oxygen saturation in a small open trial from China.^16^ In a prospective observational study from Italy, 100 patients with rapidly progressive respiratory failure, refractory to pharmacological therapy and ventilatory support were treated with tocilizumab.^17^ All patients receiving intravenous tocilizumab were also on a standard pharmacological protocol, including antiviral drugs (lopinavir 400 mg + ritonavir 100 mg twice a day or remdesivir 100 mg/day), antibiotic prophylaxis (azithromycin, ceftriaxone or piperacillin/tazobactam), hydroxychloroquine 400 mg/day and dexamethasone 20 mg/day. At 24–72 h after tocilizumab administration, 58 patients (58%) exhibited rapid improvement of their clinical and respiratory condition, 37 (37%) stabilized compared to the rapidly declining pre-tocilizumab condition, and 5 (5%) worsened (of whom 4 died). At 10 days, the respiratory condition was improved or stabilized in 77 (77%) patients, of whom 61 showed significant clearing of diffuse bilateral opacities on chest x-ray and 15 were discharged from the hospital. Respiratory condition worsened in 23 (23%) patients, of whom 20 (20%) died. The observational nature of the study and the lack of control group are significant limitations.

In another observational study based on the SMAtteo COvid19 REgistry (SMACORE),^18^ tocilizumab was compared to standard of care in a retrospective manner. Primary outcomes included ICU admission and 7-day mortality rate. Propensity score matching was employed to minimize differences between patients in the different treatment groups. A total of 112 subjects were included in this analysis. Of these patients, 21 (18.75%) received tocilizumab, whereas 91 (81.25%) patients received standard of care. Treatment with tocilizumab did not significantly affect ICU admission and 7-day mortality rate when compared with standard of care.

More recently, in an open-label, randomized controlled trial, the French CORIMUNO-TOCI trial, patients were selected on the basis of being hospitalized for COVID-19 moderate or severe pneumonia not requiring intensive care upon admission. The primary composite outcome was need for ventilation (non-invasive or mechanical) or death at day 14. A total of 129 patients were randomized: 65 to standard of care + tocilizumab and 64 to standard of care alone. Although results of this study have not yet been officially published, it was reported that a significantly lower proportion of patients reached the primary outcome in the tocilizumab arm.^19^ There are currently several ongoing trials examining the role of IL6 and IL6R blockade in a randomized controlled trial setting.

Another potent cytokine, IL1 has been tested as a potential therapeutic target in severe COVID-19. IL-1 blockade with rhIL-1Ra (anakinra) has proven efficacious in a wide array of medical conditions associated with cytokine storm. The Ana-COVID study compared a prospective cohort of patients who received anakinra to a historical control group who received standard of care.^20^ 52 consecutive patients were included in the anakinra group and were compared to 44 historical patients. Patients had severe COVID-19-related bilateral pneumonia and critical pulmonary function defined by oxygen saturation of <93% under ≥6 L/min of oxygen or oxygen saturation of <93% on 3 L/min with a saturation on ambient air decreasing by 3% in the previous 24h. Anakinra was used at a dose of 100 mg twice daily for 72h, followed by 100 mg daily for 7 days. Patients also received oral hydroxychloroquine 600 mg/day for 10 days, oral azithromycin 250 mg/day for 5 days, and parenteral β-lactam antibiotics (intravenous ceftriaxone 1g per day or intravenous amoxicillin 3g per day) for 7 days, in the absence of respective contraindications. All patients received thromboembolic prophylaxis. No oral corticosteroids or vasopressors were used, but some patients received an intravenous bolus of methylprednisolone (500mg). Anakinra significantly reduced both need for invasive mechanical ventilation in the ICU and mortality among patients with severe COVID-19, without serious side-effects. Randomized controlled trials further exploring the role of IL-1 inhibition are ongoing.

High dose of corticosteroids is effective in suppressive inflammation in hyperinflammatory states, alone or in combination with more targeted treatments. It was previously shown that corticosteroid use in patients with SARS, MERS, and influenza was associated with no survival benefit and possible harm (eg, delayed viral clearance, avascular necrosis, psychosis, diabetes). Retrospective observational data in 201 patients with confirmed COVID-19 pneumonia who developed ARDS showed that methylprednisolone appeared to reduce the risk of death.^21^ Among patients with ARDS, of those who received methylprednisolone treatment, 23 of 50 (46%) patients died, in comparison to 21 of 34 (61.8%) who did not. An open-label, multicentre, randomized controlled study was recently completed in China and investigated the use of methylprednisolone in conjunction with standard care in patients with confirmed COVID-19 infection that progressed to acute respiratory failure; results have not yet been published.^22^ Hitherto, the recommendations advise against use of corticosteroids in the treatment of early or mild disease since the drugs can inhibit immune response, reduce pathogen clearance, and increase viral shedding. NIH recommends against the routine use of systemic corticosteroids for the treatment of COVID-19 in hospitalized patients unless they are in the intensive care unit.

Finally, early observations showed that significant coagulopathy with elevated D-dimer, lactate dehydrogenase and fibrinogen and clinical thromboembolic manifestations, such as pulmonary emboli, are common features of severe COVID-19. Zhang et al.^23^ reported significant coagulopathy with multiple infarctions accompanied by prothrombotic antiphospholipid antibodies in three cases of COVID-19. Endothelial damage is a prominent manifestation in COVID-19 and can initiate thrombotic microangiopathy (TMA), which contributes to mortality, as reported in COVID-19 autopsy studies. Patients with disseminated intravascular coagulation and TMA frequently exhibit complement activation and share the clinical consequences of thrombocytopenia, microangiopathic haemolytic anaemia and microvascular thrombosis. A number of disorders such as paroxysmal nocturnal haemoglobinuria and more recently even catastrophic antiphospholipid syndrome are driven by complement and may be termed ‘complementopathies’.^24^ Complement inhibition improves significantly the course and prognosis of these diseases. Severe COVID-19 could be considered a complementopathy and complement inhibition could be considered as a treatment approach, something that is currently being tested also in randomized controlled trials.^25^

There are several significant challenges pertaining to the use of immunomodulation in severe COVID-19. The first is to be able to identify the right group of patients who would truly benefit from such an approach. Then, having predictors of response are of paramount importance. In parallel to that, the correct timing of immunomodulation of the inflammatory reaction is crucial in avoiding excess suppression of the anti-viral host response. The medical community still has a lot to learn in this fight.

## CONCLUSION

During the last few months, medicine has faced an unprecedented challenge in multiple levels. As we are all in the proverbial same boat hurled in the COVID-19 storm, we should remember that in order to stay afloat perhaps the most important tool is to join forces with colleagues and collaborate with physicians, managers and politicians. Apart from the important role that we have as rheumatologists to serve on the front lines in this pandemic setting and ensure the continuous care of our own vulnerable patient population, we also have a duty to contribute with our deep immunological knowledge and clinical experience with immunomodulatory treatments to the development of scientific evidence through good research regarding optimal treatment strategies against COVID-19. That is the new goal that life has put on us in the wake of the new decade.

“What’s true of all the evils in the world is true of plague as well. It helps men to rise above themselves.”

— Albert Camus, *The Plague*
